# The impact of smartphone-dispatched CPR-trained volunteers on OHCA outcomes is influenced by patient age

**DOI:** 10.1038/s41598-024-81263-8

**Published:** 2024-11-29

**Authors:** Johanna Fabianek, Marc Felzen, Kim R. Riester, Stefan K. Beckers, Rolf Rossaint, Hanna Schröder, Mark Pitsch

**Affiliations:** 1 Department of Anesthesiology, Medical Faculty, RWTH Aachen University, University Hospital RWTH Aachen, Pauwelsstraße 30, Aachen, 52074 Germany; 2 Aachen Institute for Rescue Management and Public Safety, City of Aachen and University Hospital RWTH Aachen, Stolberger Straße 155, Aachen, 52068 Germany; 3 Medical Direction of Aachen Fire Department, Stolberger Straße 155, 52068 Aachen, Germany

**Keywords:** Out-of-hospital cardiac arrest, Resuscitation, Resuscitation-free interval, First responder system, Mobile-phone, Smartphone-based alerting system, Health care, Preclinical research

## Abstract

The early initiation of cardiopulmonary resuscitation (CPR) measures by non-professionals before the arrival of Emergency Medical Service (EMS) is known to be crucial for improving outcomes after out-of-hospital cardiac arrest (OHCA). We assessed the impact of deploying CPR-trained volunteers via a smartphone-based alerting system on the outcome of OHCA patients. In a retrospective nonrandomized cohort study, all OHCA cases in the city of Aachen over a six-year period were analysed. We compared patient data, CPR metrics, alerting system data as well as outcome data between the intervention and control groups. From June 2017 to May 2023, 101 out of 852 resuscitations were initiated by volunteers alerted via a smartphone-based alerting system in OHCA events. We found no overall rise in the return of spontaneous circulation (ROSC) rate. An age-dependent subgroup analysis indicated an increased incidence of initially shockable rhythms and an increased ROSC rate for patients younger than 60 years in the intervention group, while implying a lower ROSC rate in patients older than 80 years after mobile responder CPR. Although this study was underpowered to yield statistically significant results, our findings suggest the need for an age-sensitive approach when evaluating the effects of first-responder systems on OHCA cases.

## Introduction

In the event of out-of-hospital cardiac arrests (OHCA), patient survival depends significantly on the no-flow-time before the initiation and during chest compressions^1,2^. In particular, a patient suffering OHCA benefits if resuscitation is started before the arrival of the Emergency Medical Service (EMS) and an AED is deployed for early defibrillations^3,4^. In Germany, the EMS takes an average of around 9 min to arrive at the scene^5,6^. The rate of bystanders performing CPR during this time interval has remained constant at around 40% for the past years despite various initiatives to promote lay CPR among the general public^5–8^. Since bystanders are usually lay helpers who are not regularly trained in CPR, resuscitation is generally less effective compared to medical professionals^9^. To shorten the interval in which the patient is not or inadequately resuscitated, various smartphone-based alerting systems have been implemented. These systems alert CPR-trained volunteers near an OHCA site and direct them to the patient, thereby increasing the chance of sufficient resuscitation measures to be carried out before EMS arrival^10,11^. Recently a large multicenter study demonstrated improvements in 30-day survival rates following the implementation of volunteer responder systems^12^.

In 2017, the smartphone-based alerting system “Corhelper” (Umlaut telehealthcare, Aachen, Germany) was introduced in the city of Aachen, Germany. To assess the impact of the implemented system on the outcome of patients with OHCA, all resuscitations initiated by CPR-trained volunteers alerted via the “Corhelper” alerting system (from here on termed “mobile responder”) were compared to those in which no mobile responder was on site to start CPR before EMS arrival. With this study, we aim to gain a better understanding of patient age as an underemphasized factor affecting the benefit of mobile responders on survival after OHCA, potentially influencing future first responder system designs and alerting strategies.

## Methods

### Study design and setting

This study covers the period of June 2017 to May 2023 from the city of Aachen, Germany. Aachen is home to approximately 262,000 inhabitants, occupying an area of 160.85 km^2^, with a population density of 1,629 inhabitants per km^2^ in mainly urban areas^13^. OHCA events in which resuscitation measures were performed were included in the study. No distinction was made regarding the reason for the cardiac arrest or patient age. Cardiac arrest scenarios in which the collapse event was witnessed by EMS personnel were excluded from this study. Through a retrospective data analysis, we examined whether the simultaneous alert of qualified volunteers via a smartphone-based alerting system and the EMS in case of an OHCA leads to an improved outcome due to the potentially earlier initiation of CPR. Therefore, we compared outcome parameters of patients who were initially resuscitated by a mobile responder with those of patients who were initially resuscitated by EMS personnel.

### EMS-response in the case of OHCA

Mission keywords indicating a potential OHCA event include “resuscitation” and "unconscious person – abnormal breathing". Before April 2021, this was only expressed by the keyword “resuscitation”. After one of these keywords is selected by the dispatch center, the conventional EMS response for potentially life-threatening emergencies involves dispatching two independent vehicles simultaneously to the emergency site. Those two vehicles typically consist of an ambulance with two paramedics and a physician-staffed vehicle with a paramedic and an emergency physician. This Franco-German two-tier system is referred to as the “rendezvous system”. At the emergency site, the emergency physician holds overall medical responsibility and makes all decisions regarding medical treatment. These decisions are based on the ERC guidelines for resuscitation, but also consider local regulations and resources as well as individual patient factors.

### Mobile responder alerting system and participants

The application “Corhelper” is a smartphone-based alerting system that notifies nearby CPR-trained volunteers within a 500-m radius of a presumed circulatory arrest. For this purpose, the resource “mobile responder” is defined within the EMS dispatch center and is simultaneously alerted when the keyword “resuscitation” or "unconscious person – abnormal breathing" is selected for a mission.

**Fig. 1:**
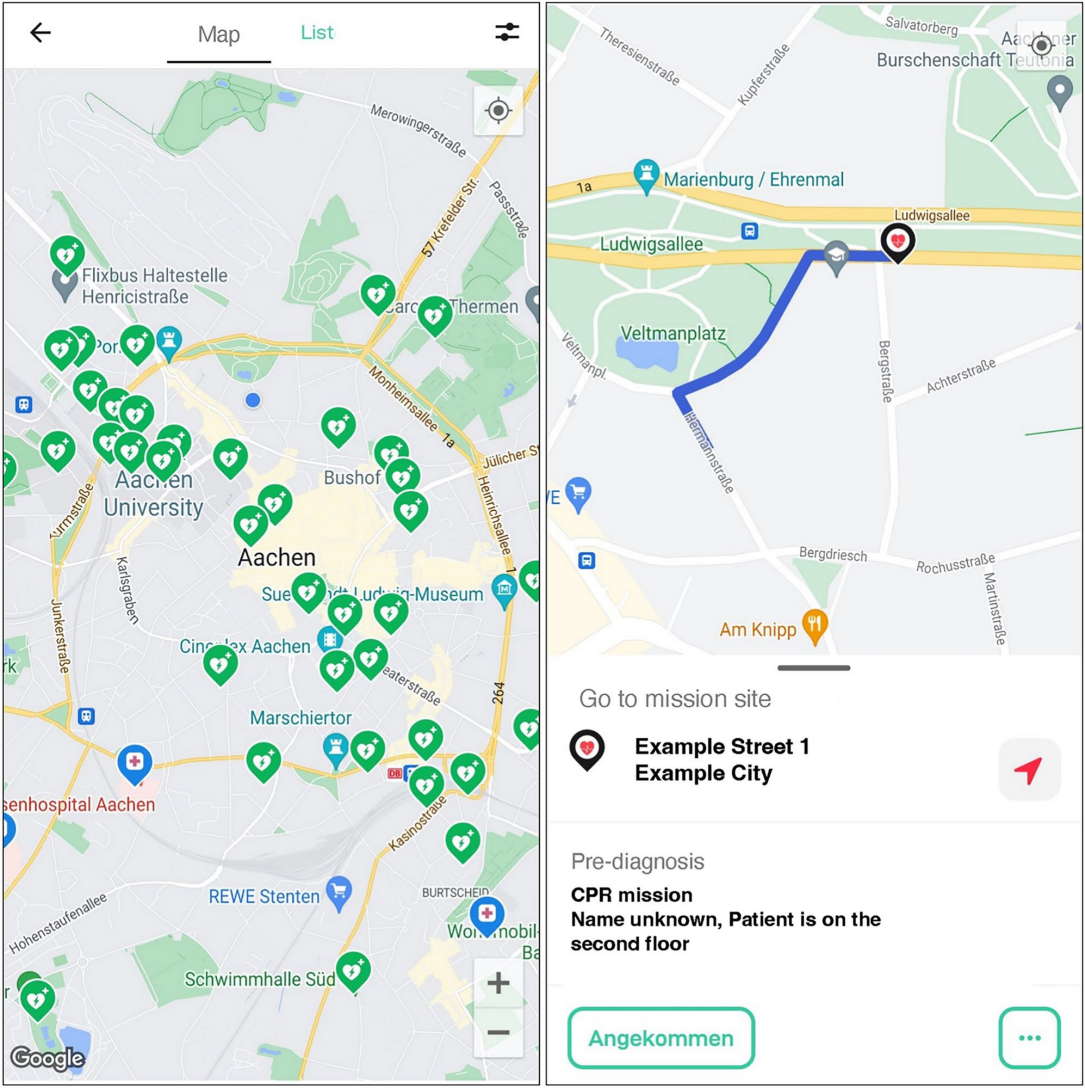
Screen of the “Corhelper” application showing the map of the city center of Aachen with available AEDs (left) and simulated “Corhelper” operation with directions to the patient (right).

In the event of an emergency alert, mobile responders can choose to accept or decline the mission. If they accept, they are navigated to the patient’s location by the smartphone application (Fig. 1). Up to three mobile responders can accept an alert, with the first two directed straight to the patient and the third retrieving an AED before being routed to the OHCA site. Upon arrival at the patient, the mobile responders indicate in the system that they have arrived. After the mission is completed, the mobile responder is asked to fill out a questionnaire about the mission. Detailed information about the alerting system can be found in the supplements (Table A.1).

**Table 1:** Baseline characteristics and overall outcome parameters of OHCA scenarios in which CPR was initiated by a mobile responder or by EMS.

	Mobile responder-initiated CPR	EMS-initiated CPR	p-value
	(n = 101)	(n = 751)	
Age in years, median [25th–75th]	73 [60–85]	72 [59–82]	0.2565
**Age in years, n (%)**			
< 60	23/101 (23%)	191/751 (25%)	
60–79	45/101 (45%)	324/751 (42%)	
> 80	33/101 (32%)	236/751 (31%)	0.8454
**Gender; n (%)**			
Female	36/101 (36%)	262/751 (35%)	
Male	65/101 (64%)	489/751 (65%)	*0.9117*
**Witness status; n (%)**			
Witnessed arrest	54/101 (53%)	410/751 (55%)	
Unwitnessed arrest	47/101 (47%)	341/751 (45%)	0.752
**Cause of cardiac arrest; n (%)**			
Cardiac	88/101 (87%)	632/751 (84%)	
Noncardiac	13/101 (13%)	119/751 (17%)	0.5576
Time to EMS arrival; (min); median [25th–75th]	*7 [6–9]*	7 [6–9]	0.3094
**Initial rhythm; n (%)**			
VT/VF	27/101 (27%)	172/751 (23%)	
PEA or asystole	74/101 (73%)	579/751 (77%)	0.3831
AED use private/public	1/101 (1%)	10/751 (1%)	> 0.9999
ROSC ever; n (%)	41/101 (41%)	314/751 (42%)	
ROSC never; n (%)	60/101 (59%)	437/751 (58%)	0.8308
**Initial outcome n (%)**			
ROSC at hospital admission	32/101 (32%)	251/751 (33%)	
Hospital admission under ongoing CPR	8/101 (8%)	74/751 (10%)	
Death on the scene	61/101 (60%)	426/751 (57%)	0.7274
RACA-Score; median [25th–75th]	35 [24.00–52.75]	35 [23.00–50.00]	0.7814
CPR = cardiopulmonary resuscitation; EMS = emergency medical service; OHCA = Out of Hospital cardiac Arrest; PEA = pulseless electrical activity; RACA = ROSC-after-cardiac-arrest; ROSC = return of spontaneous circulation; VF = ventricular fibrillation; VT = ventricular tachycardia			

### Data acquisition and classification

**Fig. 2:**
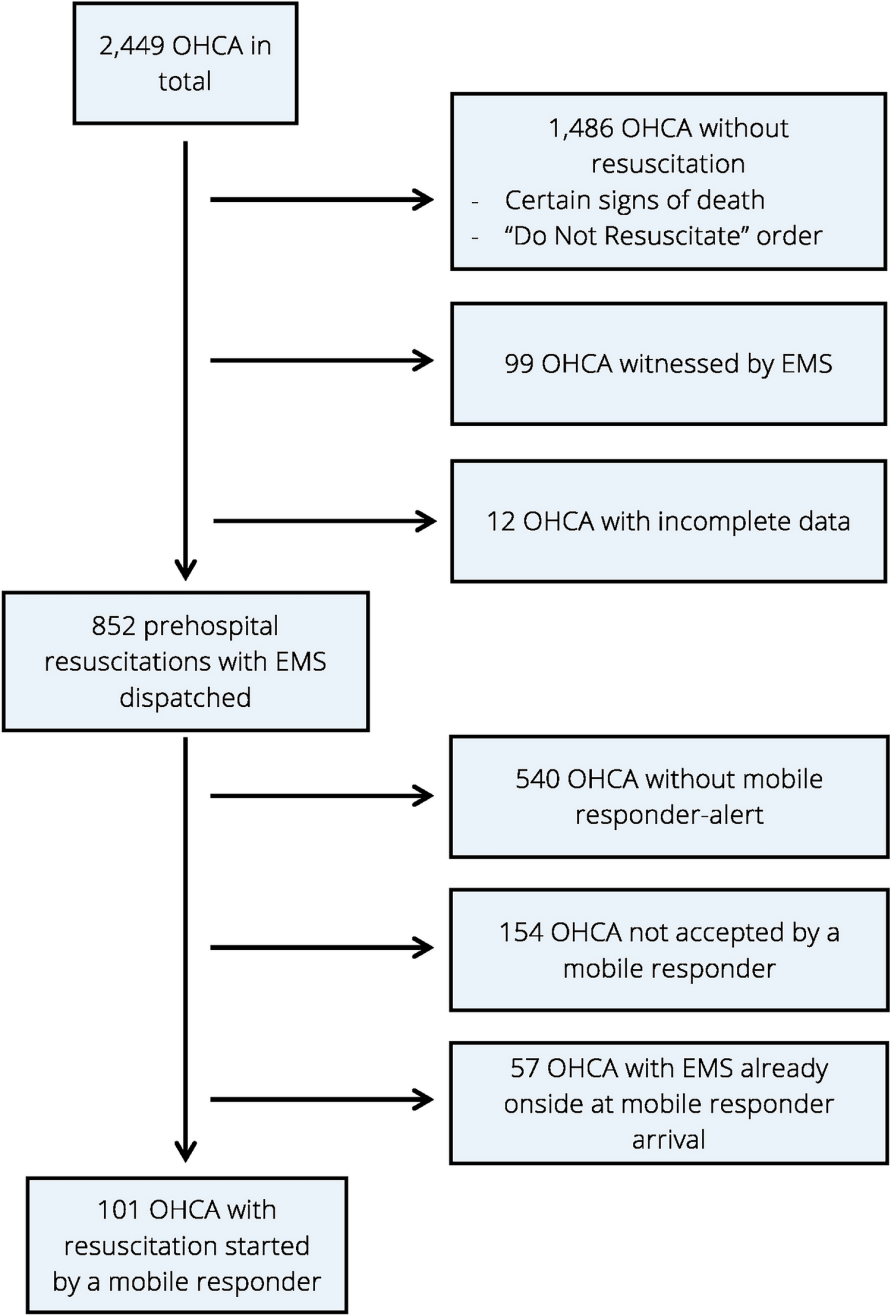
Mobile responder inclusion flowchart. *EMS* = emergency medical service;* OHCA* = Out-of-hospital cardiac arrest.

The data used for this study consist of anonymized EMS documentation of OHCA events and associated data of the dispatch center, collected according to the Utstein protocol^14^ as well as mission data from the ”Corhelper” system. EMS documentation and dispatch center data were matched based on the IDs that are specifically assigned and documented for each mission. The exact date and time of mission initiation in the “Corhelper” system allowed a clear assignment of mobile responder missions to those data. After identifying OHCA cases where no mobile responder alert was triggered or no mobile responder accepted the mission, the mission protocols of the emergency physician and the surveys completed by the mobile responders after finishing the mission were analysed to determine those cases in which the mobile responder actually initiated CPR (Fig. 2).

### Main outcomes and subgroup definitions

The primary outcome measure was the rate of return of spontaneous circulation (ROSC), while secondary outcomes included the initial ECG rhythm upon EMS arrival and the patient’s condition upon hospital admission. ROSC was defined as the presence of an organized rhythm in the ECG together with a palpable central pulse as documented in the protocol of the emergency physician, possibly accompanied by clinical indicators such as spontaneous movements, breathing activities or a sharp increase in end-tidal CO2.

For a more detailed analysis, patients were categorized into subgroups based on their age. Furthermore index patients were defined as those with the highest expected outcome^2,15^, characterized by ventricular fibrillation as the initial rhythm, a witnessed cardiac arrest, and a cardiac cause for the arrest.

### Comparisons with the RACA score

To evaluate the likelihood of achieving ROSC in each patient, the ROSC-after-cardiac-arrest (RACA) score was calculated and compared to the observed ROSC rate. This statistical parameter was developed by Gräsner et al. in order to predict the likelihood of ROSC in individual OHCA cases. The score considers eight different parameters, which are factored into a formula with either a positive or negative influence, ultimately providing the likelihood of ROSC in this particular case^16^. Details about the underlying variables and the calculation of the RACA score within the German Resuscitation Registry can be found in the supplements (Table A.2). Practically the RACA score is usually not applied for individual patient care on scene, but rather used to monitor and benchmark the performance of EMS systems retrospectively. For this purpose, the RACA score has been validated in different regions around the world^17,18^. In the interest of a valid comparison of ROSC rates, it allows for an adjustment based on baseline conditions for successful resuscitations in each individual emergency service area. In the present study, we used the RACA score to compare the expected and the observed ROSC rates in the age-depended subgroups described above.

**Table 2:** Baseline characteristics and outcome parameters of OHCA scenarios in which CPR was initiated by a mobile responder or by EMS separated in patients under 60 years.

	Mobile responder-initiated CPR	EMS-initiated CPR	p-value
Patients under 60 years, n	23	191	
ROSC ever; n (%)	15/23 (65%)	97/191 (51%)	
ROSC never; n (%)	8/23 (35%)	94/191 (49%)	0.2691
ROSC at hospital admission; n (%)	12/23 (52%)	78/191 (41%)	
Hospital admission with ongoing CPR; n (%)	7/23 (30%)	34/191 (18%)	
Death on the scene; n (%)	4/23 (17%)	79/191 (41%)	0.0679
RACA-Score; Median [25th–75th]	45.5 [24.50–62.75]	38 [27.00–59.75]	0.4254
**Initial rhythm; n (%)**			
VT/VF	11/23 (48%)	60/191 (31%)	
PEA or asystole	12/23 (52%)	131/191 (69%)	0.158
**Witness status; n (%)**			
Witnessed arrest	14/23 (61%)	105/191 (55%)	
Unwitnessed arrest	9/23 (39%)	86/191 (45%)	0.6610
CPR = cardiopulmonary resuscitation; EMS = emergency medical service; OHCA = Out of Hospital cardiac Arrest; PEA = pulseless electrical activity; RACA = ROSC-after-cardiac-arrest; ROSC = return of spontaneous circulation; VF = ventricular fibrillation; VT = ventricular tachycardia			

### Statistical analysis

Continuous variables were presented as median and interquartile range (IQR; 25th–75th percentile), and categorical variables were presented as absolute numbers and percentages. To test for statistical significance, the Fisher exact test and Chi-square test were conducted to analyse differences in proportions in the case of categorical data, and Wilcoxon’s rank-sum test was used to compare unpaired groups of continuous variables. Multiple logistic regression analyses were conducted to evaluate interactions within sub-groups. A p-value of < 0.05 was considered statistically significant. All statistical analyses were performed using GraphPad Prism version 10.0.1 for Windows, GraphPad Software, Boston, Massachusetts, USA (www.graphpad.com).

### Ethics approval and consent to participate

The Ethics Committee at the RWTH Aachen Faculty of Medicine (Pauwelsstraße 30, 52074 Aachen, Germany) approved the analysis without any constraints (approval number: 109/15). The need for informed consent was waived by the Ethics Committee at the RWTH Aachen Faculty of Medicine (Pauwelsstraße 30, 52074 Aachen, Germany, Head: Prof. Hausmann), the Center for Translational and Clinical Research (CTC-A) of RTWH Aachen University and the responsible data protection officers, because this retrospective analysis was performed anonymously in the context of legally required quality assurance under the responsibility of municipal authorities. All research was performed in accordance with relevant guidelines and regulations.

## Results

### Progression of mobile responder alerts and increase in the number of mobile responders

There was a steady increase in the number of registered participants in the smartphone-based alerting system, from 548 mobile responders in 2017 to 1,953 mobile responders in 2023. Concurrently, the recognition of OHCA scenarios resulting in system activation increased considerably, from 19% in 2017 to 56% in 2023, with a sharper increase since 2021 after adjusting the critical mission keywords (Fig. 4, Table A.3).

### Mission characteristics and overall outcome data

Between June 2017 and May 2023, 2,449 OHCA occurred in the city of Aachen. In 852 cases, the EMS was dispatched to a cardiac arrest scenario. Out of these, the mobile responder system was activated in 312 cases. However, in the remaining 540 cases, the cardiac arrest was not recognized initially based on the emergency call (Table A.4, Data Supplements). In 158 cases in which the system was activated, at least one mobile responder accepted the mission. Of these, 57 arrived at the scene after the EMS. This resulted in 101 resuscitations initiated by mobile responders and 751 initiated by the paramedics or emergency physicians (Fig. 2). Patient and resuscitation characteristics are shown in Table 1.

EMS arrived after an average of 7 (6–9) minutes for both resuscitations started by mobile responders and resuscitations started by EMS. On average, the patients demonstrated the same RACA values (35 in both groups, p = 0.7814). Overall, patients who were resuscitated by a mobile responder before the arrival of the EMS did not have a higher ROSC rate (41% vs. 42%, p = 0.8308). The witness status and incidence of cardiac causes of OHCA showed no differences in both groups. Patients were defibrillated before the arrival of EMS in 1% of cases either by a mobile responder in the intervention group or by lay bystanders in the control group. After the initiation of CPR by a mobile responder, patients presented a higher incidence of shockable rhythms in their initial ECG (27% vs. 23%; p = 0.3831).

### Patient population separated into age groups

**Fig. 3:**
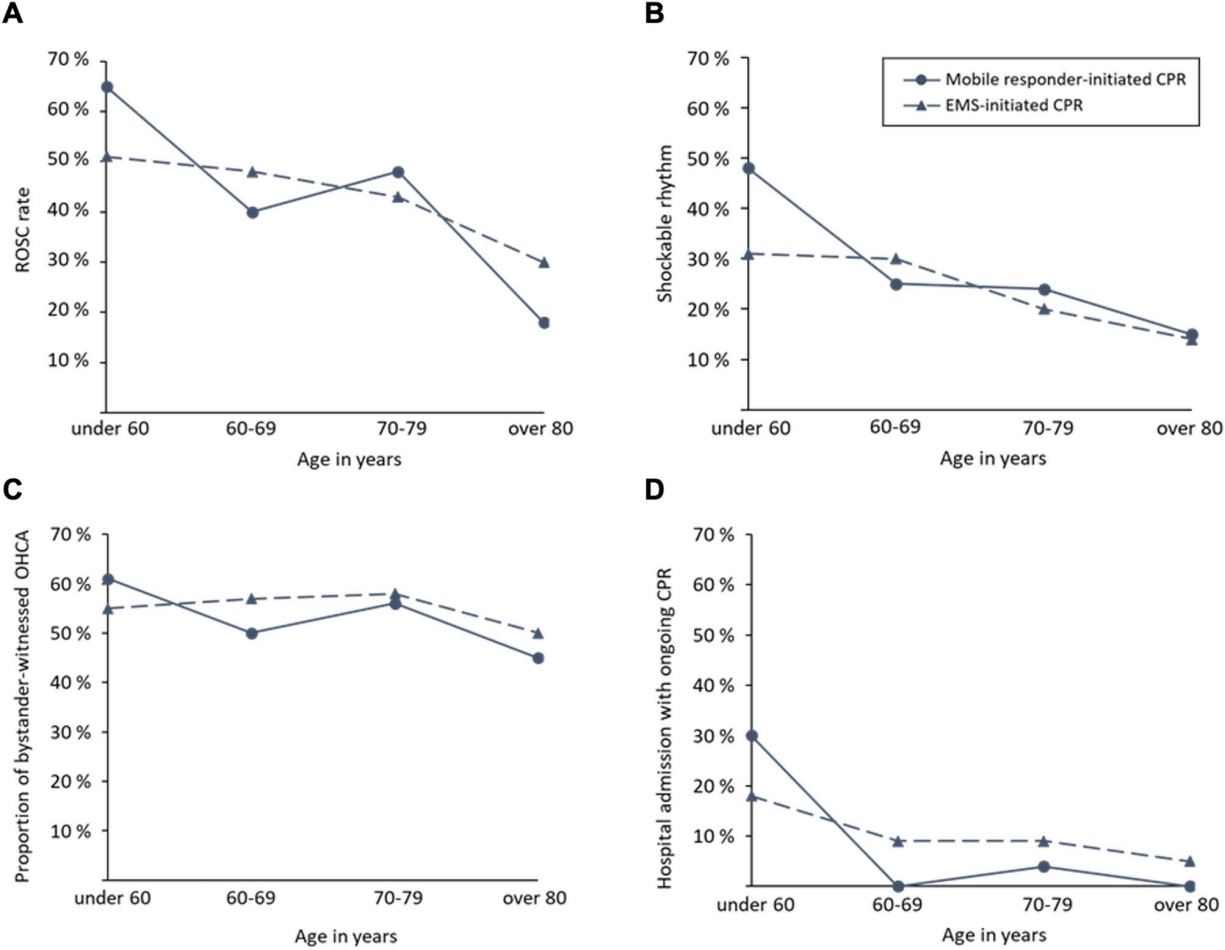
Comparison between mobile responder-initiated resuscitations and EMS-initiated resuscitations regarding outcome parameters in different age groups. (**A)** ROSC-rate, (**B**) Initial shockable rhythm, (**C**) Proportion of bystander-witnessed OHCA, (**D**) Proportion of hospital admission with ongoing CPR. *EMS* = emergency medical service;* OHCA* = Out-of-hospital cardiac arrest;* CPR* = cardiopulmonary resuscitation.

To gain a more differentiated insight into patient characteristics, we separated the patients into age groups (Table 2 and Table A.5; Data Supplements). In the group of patients under 60 years, the ROSC rate was higher in cases where CPR was initiated by a mobile responder (65% vs. 51%; p = 0.2691). Notably, in patients older than 80 years, initiation of CPR by a mobile responder resulted in a lower ROSC rate (18% vs. 30%; p = 0.2168; Fig. 3a). An initially shockable rhythm was more common in patients under 60 years following mobile responder-initiated CPR (48% vs. 31%; p = 0.1580; Fig. 3b). There was no relevant difference in the amount of observed cardiac arrests (Fig. 3c). The proportion of patients admitted to the hospital during ongoing resuscitation was 30% among patients younger than 60 years for mobile responder-initiated CPR and 18% for resuscitations initiated by EMS, resulting in 17% of patients dying on scene after being resuscitated by mobile responders and 41% for resuscitations initiated by EMS (p = 0.0679; Fig. 3d). Multiple logistic regression analyses were performed to evaluate the association between Corhelper involvement and ROSC in different age groups. They showed moderate discriminative ability with an AUC of 0.6118 (95% CI: 0.57 to 0.65, p < 0.0001) and confirmed the aforementioned trends without reaching statistical significance. Results indicate a 52% increase in the odds of ROSC in patients younger than 60 years (OR 1.52 [95% CI: 0.45, 5.39]) and a decrease of 56% of the odds of ROSC in patients older than 80 years (OR 0.44 [95% CI: 0.13, 1,50]) in the case of mobile responder initiated CPR.

Comparisons with the RACA-score consistently showed that the observed ROSC rates were consistently higher than predicted, indicating overall good OHCA management.

Noticeably, there was a difference in the case of patients younger than 60 years. We observed a ROSC rate of 65% compared to a RACA-score of 45.5 after mobile responder-initiated CPR and a ROSC rate of 51% compared to a RACA-score of 38 after EMS-initiated CPR in this age group. Only in the case of patients older than 80 years who were initially resuscitated by a mobile responder the ROSC rate was lower than predicted (18% compared to a RACA score of 27).

### Index patients

The proportion of index patients in the group of resuscitations started by mobile responders was 23% compared to 17% in the control group. The ROSC rate in both groups was comparable at 74% and 76%, and there was no relevant difference in hospital admission rates (Table A.6; Data Supplements).

## Discussion

In this observational study, conducted over a six-year period, we investigated the effects of volunteers trained in resuscitation who were alerted via a smartphone-based alerting system on patients with OHCA. In 12% of resuscitations performed in this period, a mobile responder initiated resuscitation measures. Our observations indicate an increase in the rate of ROSC and the occurrence of initially shockable rhythms in OHCA patients under 60 years who were resuscitated by a mobile responder. Combined with a trend towards an increased rate of transportation under ongoing resuscitation, this resulted in a noticeable decrease in deaths at the scene in this age group. Even though these results did not reach the threshold of statistical significance, they should still be considered when evaluating the outcomes of first responder alert systems.

Patients who initially received resuscitation from either mobile responders or EMS presented comparable baseline characteristics in terms of gender, age distribution, RACA-score, witnessed status, and EMS arrival time, ensuring good comparability. Both groups had a ROSC rate of about 41% and showed no significant differences in the occurrence of shockable rhythms or hospital admission rates. Similar studies evaluating different comparable smartphone-based alerting systems have been conducted on this topic in recent years, most of which have yet to show a clear survival benefit^19–21^. In the case of those studies that show an increased ROSC rate^10,22,23^, there are some relevant differences to our study regarding the patient population^20^ or the study design^21^. We believe that another potential reason for the lack of an improved overall ROSC rate in our study is the short EMS arrival time in the city of Aachen of around 7 min in comparison to similar studies investigating smartphone-based alerting systems^10,20,21,23^. This results in only a narrow time gap in which the efforts of the mobile responder can actually create a measurable benefit for patients on a large scale.

Patients under 60 years showed an increased rate of ROSC (65% vs. 51%), a higher rate of shockable rhythm (48% vs. 31%) and were more frequently admitted to the hospital under ongoing CPR (30% vs. 18%) when initially resuscitated by a mobile responder. Less distinctive and without division into age groups, other studies also showed that patients resuscitated by mobile responders tended to have a shockable initial rhythm^10,23^, presumably because earlier resuscitation maintains ventricular fibrillation longer^25,26^. Another noticeable feature is the markedly higher proportion of patients under 60 years of age resuscitated by mobile responders who were admitted to hospital under ongoing resuscitation.

In patients older than 60 years, we no longer observe a difference in the occurrence of shockable rhythms, while we even find a lower rate of ROSC in cases of mobile responder-initiated resuscitations in patients older than 80 years. We suspect that the reason for this observation is that mobile responders initiate many resuscitations that would not be started by EMS. Mobile responders are instructed to initiate resuscitation without actively checking for extensive comorbidities, certain signs of death, or evaluating a possible “Do Not Resuscitate” status of the patient, which could be reasons for the EMS not to start resuscitation in the first place. These situations are more common in the elderly.

### Consequences and practical implementations

**Fig. 4:**
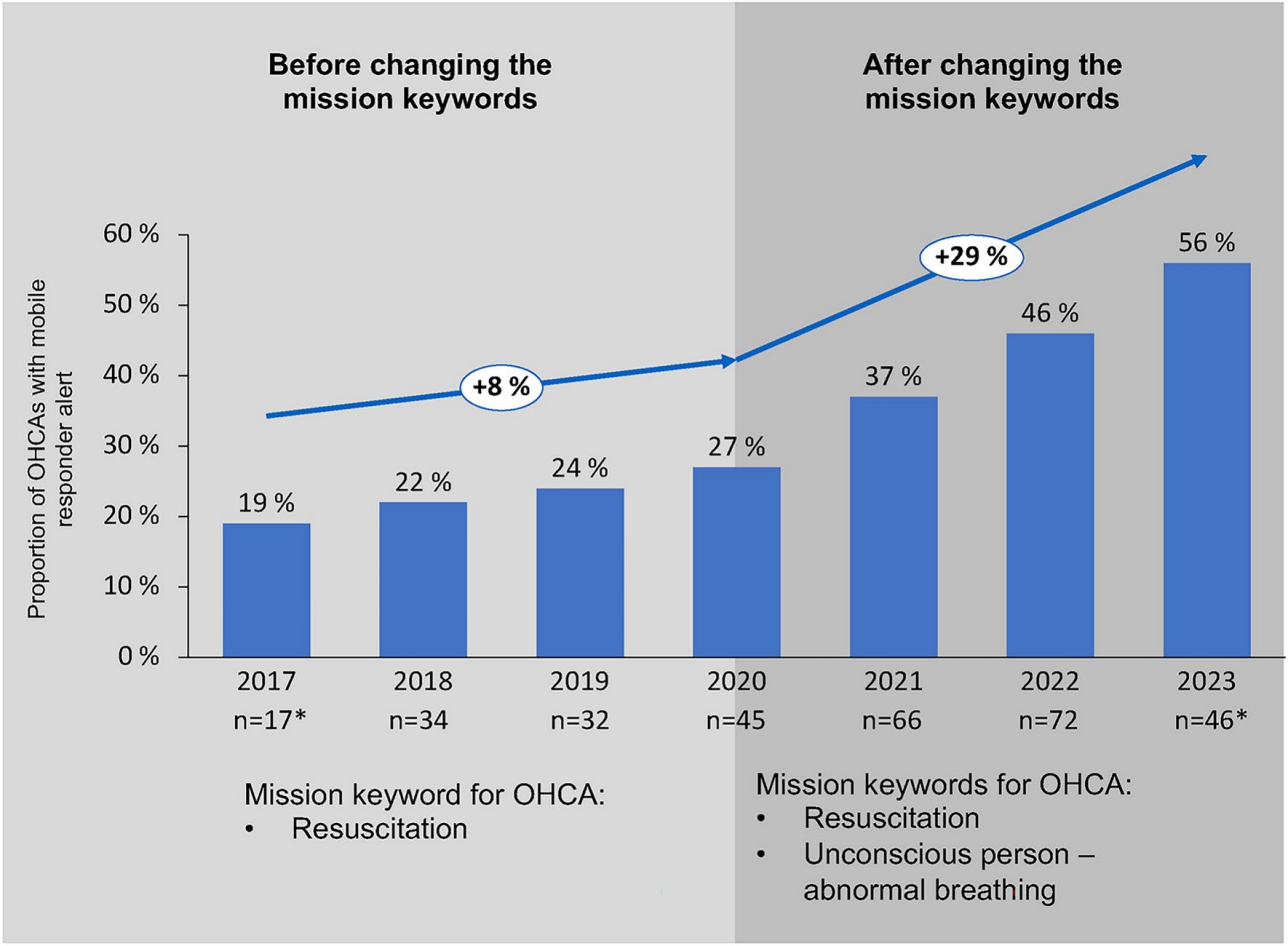
Proportion and absolute numbers of mobile responder alerts among all OHCAs in 2017 and 2023. *OHCA* = Out-of-hospital cardiac arrest.

Based on these findings, it is imperative to continue advocating for lay resuscitation, increase the registration of individuals as mobile responder to extend mobile responder coverage, and enhance the identification of cardiac arrest scenarios in emergency calls. In April 2021, the mission keyword "unconscious person – abnormal breathing" was introduced as an additional trigger for system activation, resulting in a noticeable increase in mobile responder alerts (Fig. 4). We suspect that this can be attributed to a higher quality of emergency call interrogation, as the sole mission keyword “unconscious person” was no longer an option. A selection between the obligatory addendum “normal breathing” or “abnormal breathing” required actively assessing the patient’s respiratory activity. Considering that an unconscious patient with abnormal breathing is highly indicative of cardiac arrest, this approach presumably contributed to the identification of resuscitation scenarios that would otherwise have been categorized as “unconscious person.”

The reliable recognition of OHCA with immediate initiation of CPR measures, preferably in combination with an early defibrillation, can improve the outcome after OHCA^27–20^, especially in young patients^31^. Since one main finding of our present study is the increased rate of initially shockable rhythms in patients resuscitated by mobile responders, it is crucial to equip bystanders and mobile responders with means to apply early defibrillations to convert an increased numbers of shockable rhythms into an increased number of ROSCs, ideally even before EMS arrival^32^.

To achieve this objective, a set of initiatives has been put into motion to increase the number of AEDs available around the clock in the city of Aachen. This includes efforts such as installing AEDs outside public buildings rather than confining them to interior spaces. Hence, the current system, in which the first two mobile responders accepting the call are directed straight to the patient and the third mobile responder serves as the AED provider, is recommended to be maintained^32^ and could, in the future, be supplemented by the delivery of AEDs via drones^33,34^.

#### Limitations

Particularly during the initial phase of this single-center study, the number of registered mobile responders was relatively low, leading to the inclusion of only 101 cases of mobile responder-initiated CPRs over the six-year examination period. This limited sample size renders this study underpowered to demonstrate statistically significant results. The findings of this study can not necessarily be transferred to rural areas, where on the one hand the EMS has longer arrival times and on the other hand the density of possible mobile responders is lower^13^. Likewise, the transfer to other EMS systems, which may operate without emergency physicians and solely rely on paramedics, demands further evaluations and specific adjustments.

This study did not investigate the time interval between the alerting time of the mobile responders and their arrival time at the scene. The determination of the mobile responders ‘ arrival is contingent on their active indication in the alerting system, potentially introducing inaccuracies. Incorporating GPS positioning and time logging could prove beneficial in addressing these potential discrepancies. Resuscitation measures started by lay bystanders were not specifically assessed as the focus of this study was the additional benefit of volunteers trained in CPR, who, in contrast to lay bystanders, are not yet part of the established rescue system and data regarding the frequency and the quality of lay bystander CPR remain poor.

## Conclusion

The effects of a smartphone-based alerting system for CPR-trained volunteers on the outcome in OHCA cases is influenced by patient age. We found indications that younger patients, in particular, benefit from an early resuscitation by mobile responders. There was a trend for improved initial conditions upon EMS arrival with an increased chance of ROSC and admission to hospital under ongoing resuscitation. The higher frequency of initially shockable rhythms underscores the need for greater involvement of AEDs within the system. A larger number of registered mobile responders appears necessary to achieve significant survival benefits in urban areas with low EMS arrival times, while further studies are needed to assess those benefits in rural areas.

## Supplementary Information

Supplementary Information.

## Data Availability

The datasets analyzed in the current study are not publicly available because they are municipal property and cannot be published online under open-access agreements. However, these datasets are available upon reasonable request and with permission from municipal authorities.

## Abbreviations

CPR: Cardiopulmonary resuscitation
OHCA: Out-of-hospital cardiac arrest
EMS: Emergency medical service
ROSC: Return of spontaneous circulation
AED: Automated external defibrillator
RACA: ROSC-after-cardiac-arrest
ECG: Electrocardiogram
IQR: Interquartile range
eCPR: Extracorporeal cardiopulmonary resuscitation
GPS: Global Positioning System
