# Supplementary material for: The impact of smartphone-dispatched CPR-trained volunteers on OHCA outcomes is influenced by patient age
Source: Sci Rep. 2024 Nov 29;14:29671. doi: 10.1038/s41598-024-81263-8 (PMC11607460; doi:10.1038/s41598-024-81263-8)
Supplement: Supplementary file 1 — Supplementary Information. [file 41598_2024_81263_MOESM1_ESM.pdf]

# Supplementary Material

## Table A.1.a

Recommended reporting items associated with first responder systems based on Müller et al.[35]. Note that the data given only refer to the use of the first responder system in the city of Aachen, Germany, since this area is included in this specific study. The first responder system itself is active in different cities and regions of Germany, where the variables that are specified in this overview may deviate.

| ***Category*** | ***Item*** | ***Description*** |
|---|---|---|
| *1A: Geospatial* | *1: Name of the region* | *Germany, city of Aachen* |
| | *2: Square km covered* | *160.85 km²* |
| | *3: Inhabitants* | *262,000* |
| | *4: OHCA incidence* | *62.8 (2022)* |
| *1B: System description* | *1: Name of the app* | *corhelper* |
| | *2: Who runs the system? Profit/Non-profit?* | *City of Aachen* |
| | *3: Relevant authorities* | *Medical Direction of Emergency Medical Service, City of Aachen* |
| | *4: AED network/database* | *Locations of AED in the region is implemented in the app. No external AED database is used.* |
| | *5: Number of FR* | *2124* |
| *1C: Role of dispatch centre* | *1: Number of emergency dispatch centres* | *One* |
| | *2: Systems in use* | *One* |
| | *3: Activation through emergency dispatch centre* | *The system is integrated into the dispatch centre control system. It can not be triggered without dispatch centre involvement.* |
| | *4: Activation of system triggered manually/automatically* | *The system is triggered automatically if the activation criteria are fulfilled.* |
| | *5: Mission cancellation (dispatch centre)* | *The app informs the FR when the mission has been cancelled.* |
| | *6: Mission abort (FR)* | *FR can notify the dispatch centre via the app if they decide to abort a mission.* |
| *1D: Maturity of responder system* | *1: Maturity* | *The system was launched in 2017. Version 2.0 of the corhelper app was launched on July 1, 2021.* |
| *1E: Daytime* | *1: Times for system activation* | *The system is continuously active.* |
| *1F: Role allocation* | *1: Number of first responders (FR) alerted in a single mission* | *Three responders get alerted in a single mission.* |
| | *2: Roles in the system* | *2 FR go to the patient, 1 FR goes to AED* |
| *1G: Activation criteria* | *1: Trigger for activation of the system* | *The keywords "resuscitation", "unconscious person – abnormal breathing“ and “bolus event - persistent” trigger he activation of the system. Those keywords are valid since April 2021. Previously, only the keyword "resuscitation" applied.* |

| | | |
|---|---|---|
| | *2: Exclusion criteria* | *OHCA of children are explicitly no exclusion criterium. The dispatch center can prevent a system activation if a danger to the FR cannot be ruled out.* |
| *1H: Delay time to system activation* | *1: Delay time* | *Less than two seconds.* |
| *1J: Places alerted to (private, public)* | *1: Sites FR are dispatched to* | *FR are dispatched to public and private places.* |
| | *2: Sites FR are not dispatched to* | *Any sites are possible if the criteria mentioned in 1G are met.* |
| *1K: Characteristics of FRs* | *1: FR groups* | *Certified first aider (28.6%), Medical student (19.8%), Paramedic (20.4%), Emergency physician (12.4%), Physician (5.5%), Nurse (5.5%), Voluntary fire brigade (3.0%), Police (2.1%). It should be noted that some occupations may also be recorded simultaneously as first aiders for technical reasons in some regions.* |
| | *2: On duty/off duty* | *As long as they don't inform the system about being unavailable, they are available.* |
| | *3: FR duties* | *Perform chest compressions, apply AED* |
| *1L: Additional features of first responder system* | *1: Feedback form/report* | *After finishing the mission, the FR is requested to file a voluntary survey.* |
| *1M: Data reporting* | *1: How are data reported (dispatch centre, system backend, app)* | *Mission data from the dispatch centre and the app are stored in the system backend and can be retrieved for follow-up analysis and quality control* |
| *1N: Psychological protection of FRs* | *1: Psychological support* | *After completion of each mission, the FR can request a professional debriefing by selecting the provided button in the app.* |

**Table A.1.b**

Recommended items associated with the first responder network.

| ***Category*** | ***Item*** | ***Description*** |
|---|---|---|
| *2A: Training* | *1: Qualification* | *The FR must be a health care professional or a first aider who has completed a BLS course.* |
| | *2: Revalidation* | *In the case of laypersons, BLS courses must be renewed every two years.* |
| *2B: Type of FR* | *1: In-/exclusion criteria for FR* | *FR must be at least 18 years of age.* |
| | *2: Volunteer or professional* | *The system doesn't include professional responders on regular duty/units. FR are volunteers (including professional responders during off-duty times. There is no payment/compensation for FR activities.* |
| | *3: Training required for FR* | *There's no mandatory training.* |
| *2C: Availability* | *1: Location of FR* | *Stationary units (such as police stations or fire stations) or mobile units (such as police officers) are not integrated.* |
| | *2: Communities/groups* | *Only individual alerts based on distance to emergency site.* |
| *2D: Equipment* | *1: Equipment provided* | *There's no equipment provided.* |

**Table A.1.c**

Recommended items associated with the technology, algorithms, and strategies.

| ***Category*** | ***Item*** | ***Description*** |
|---|---|---|
| *3A: Technical availability* | *1: Operating system* | *The smartphone app works with android and iOS.* |
| | *2: Critical alert* | *The app offers a feature to play an alarm sound if the phone is on 'do not disturb' or 'silent' mode for both android and iOS systems.* |
| *3B: Location technology* | *1: Mobile Phone Positioning System* | *The first responders are located via mobile phone positioning system.* |
| | *2: First responder tracking* | *The first responders are not being tracked during the mission.* |
| | *3: Position updates* | *How often are positions of first responders updated?*<br>*(1) Before an alarm: every 5-10 minutes*<br>*(2) During a mission: no tracking / position updates* |
| | *4: Navigation* | *A map and navigation feature are included in the app-system.* |
| | *5: AEDs visible in app* | *The AED positions are visible in the app.* |
| *3C: Algorithm* | *1: Distance calculation* | *The linear distance between the FR and the emergency site are calculated in case of an alarm.* |
| | *2: Alerting radius* | *500 m* |
| | *3: Mode of transportation* | *The mode of transportation of the first responder is not recognized by the system.* |
| | *4: Alarm procedure* | *All FRs within the alarm radius are alerted. The first FRs to accept the alarm are selected. The first two FRs are routed directly to the patient, the third is the AED provider.* |
| | *5: Responding alarms* | *First responders who receive an alarm can accept and reject it. The data about acceptance and rejection is available in the system for research and quality management. The respective information is not visible in the dispatch centre.* |
| | *6: New technology algorithm* | *Not included.* |
| | *7: AED task* | *The system guides the third FR who accepts the mission to the closest AED.* |
| *3D: Legal* | *1: Privacy restrictions* | *There are no legal regulations limiting the (technological) feature of the alerting system.* |

**Table A.1.d**

Recommended items associated with data collection.

| Category | Item | Description |
|---|---|---|
| *4A: System data* | *1: Activation rate* | *368 alerts in 2022; 366 alerts in 2023* |
| | *2: Response rate* | 77.4% in 2022; 81.0% in 2023 |
| | *3: Number of first responders per case* | *0.75 in 2022; 0.76 in 2023* |
| | *4: First responder at the scene rate* | *0.60 in 2022; 0.60 in 2023* |
| | *5: Acceptance rate* | *0.44 in 2022; 0.44 in 2023* |
| | *6: Arrival before EMS rate* | *0.07 in 2022; 2023 not yet completely evaluated* |
| | *7: AED arrival before EMS rate* | *Unknown* |
| | *8: Distance FR - Emergency location* | *500 m airline distance* |
| *4B: Time* | *1: Call-alarm interval* | *Unknown* |
| | *2: FR response time* | *On average, the FRs need around 7 minutes to reach the patient. The method of measuring the arrival time is based on the manual input of the FR upon arrival in the app.* |
| | *3: Call-response interval AED* | *Unknown.* |
| | *4: Call-shock interval* | *Unknown.* |
| *4C: CPR cases* | *1: CPR rate* | *18% in 2022* |
| | *2: AED rate* | *Less than 1% in 2022* |
| | *3: AED shock rate* | *Less than 1% in 2022* |
| *4D: Outcome* | *1: Emergency location* | *Information not available in the system.* |
| | *2: Means of transport* | *Information not available in the system.* |
| | *3: Adverse safety event rate* | *No adverse events have been recorded yet.* |
| | *4: Need for debriefing rate* | *2 (debriefings) / 1421 (missions in total) = 0.14%* |

**Table A.1.e**

Recommended items associated with AEDs.

| Category | Item | Description |
|---|---|---|
| *5A: AED registry* | *1: Number of AEDs* | *137 AEDs in total* |
| | *2: AED network* | *There is no standard since various AEDs of different brands are in use. Usually these are semi-automatic and with chest compression feedback.* |
| | *3: AED drones* | *There are no AED drones in the system yet.* |
| | *4: Mobile AEDs* | *There are no mobile AEDs in the system.* |
| | *5: Maintenance* | *Maintenance is carried out individually by the operators.* |

| | | |
|---|---|---|
| | *6: Legal obligations* | *There are no legal obligations to register private AEDs and to make them available for first responders.* |
| | *7: Strategic placements* | *There is no strategic placement of AEDs yet.* |
| | *8: Data retrieval* | *The use of an AED is documented by the EMS involved.* |
| *5B: Mode of delivery* | *1: Delivery: FR* | *There was one case in which a stationary AED was fetched by first responder.* |
| | *2: Delivery: Drone* | *There was no case in which the AED was delivered by drone.* |
| | *3: Delivery: Mobile* | *There was no case in which a mobile AED was delivered.* |
| *5C: Accessibility and availability* | *1: Accessibility of AEDs* | *3 AEDs available 24/7* |
| | *2: AED visibility* | *The AEDs are visible in the alerting app.* |
| *5D: Post incidence report* | *1: First rhythm AED* | *Unknown.* |
| | *2: AED data* | *No data retrieval from an AED is carried out.* |
| | *3: Number of shocks* | *As reported by FR. No data retrieval from an AED is carried out.* |
| | *4: Distance AED* | *Not documented.* |

**Table A.2**

Variables that are considered in the RACA score.

| **Variable** |
|---|
| Gender |
| *Age* |
| *Cause for cardiac arrest* |
| *Witness status* |
| *Location of cardiac arrest* |
| *Initial ECG rhythm* |
| *Bystander CPR* |
| *EMS arrival time* |
| Calculation of the RACA score was performed within the German Resuscitation Registry (https://www.reanimationsregister.de/) based on the initially published formula by Gräsner et al. [15] |

**Table A.3**

Absolute number and proportion of mobile responder alerts in OHCA events, number of mobile responders in total and mobile responders per km² between 2017 and 2023.

| | 2017* | 2018 | 2019 | 2020 | 2021 | 2022 | 2023* |
|---|---|---|---|---|---|---|---|
| OHCA in total; n | 89 | 156 | 134 | 168 | 177 | 157 | 82 |
| OHCAs with mobile responder alert (%) | 17 (19%) | 34 (22%) | 32 (24%) | 45 (27%) | 66 (37%) | 72 (46%) | 46 (56%) |
| Number of mobile responders in total; n | 548 | 690 | 830 | 999 | 1481 | 1852 | 1953 |
| Increase on previous year; % | - | 26% | 20% | 20% | 48% | 25% | 5% |
| Mobile responder-initiated CPR; n (%) | 4 (5%) | 2 (1%) | 6 (5%) | 18 (12%) | 33 (22%) | 25 (18%) | 13 18%) |
| EMS-initiated CPR; n | 76 | 136 | 114 | 128 | 121 | 116 | 60 |
| ROSC in mobile responder-initiated CPR; n (%) | 3 (75%) | 1 (50%) | 2 (33%) | 7 (39%) | 11 (33%) | 13 (52%) | 4 (31%) |
| ROSC in EMS-initiated CPR; n (%) | 35 (46%) | 65 (48%) | 55 (48%) | 53 (41%) | 43 (36%) | 39 (34%) | 24 (40%) |

*In the years 2017 and 2023, the entire year is not recorded. In 2017 June to December and in 2023 January to May were analysed.

OHCA = Out of Hospital Cardiac Arrest; CPR = Cardiopulmonary resuscitation, EMS = emergency medical service

**Table A.4**

Mission keywords of not detected resuscitation events*.

| Mission keywords of not detected resuscitation events | n=540 |
|---|---|
| Unconscious person | 131/540 (24%) |
| Acute shortness of breath | 61/540 (11%) |
| Unconscious person – normal breathing | 53/540 (10%) |
| Acute coronary syndrome (ACS) | 31/540 (6%) |
| Lifeless person | 23/540 (4%) |
| Persistent seizure | 15/540 (3%) |
| Stroke | 9/540 (2%) |
| Bolus event | 5/540 (1%) |
| Bradycardia | 4/540 (1%) |
| Others (see comment) | 116/540 (21%) |
| No mission keyword | 92/540 (17%) |

*Others including trauma scenarios, potentially unsafe emergency sites (e.g. traffic accidents) and further circumstances and conditions related to a fire or emergency, necessitating the intervention of the fire department, as well as wide variety of internal/neurological disorders.*

*Note that these not detected resuscitation events potentially include situations in which the patient was still alive at the time of the emergency call, and their condition deteriorated to the point of requiring resuscitation during EMS approach.

**Table A.5.a**

Baseline characteristics and outcome parameters of OHCA scenarios in which CPR was initiated by a mobile responder or by EMS in patients between 60 and 69 years.

| | *Mobile responder-initiated CPR* | *EMS-initiated CPR* | *p-value* |
|---|---|---|---|
| ***Patients 60-69 years*** | 20 | 141 | |
| *ROSC ever; n (%)* | *8/20 (40%)* | *67/141 (48%)* | |
| *ROSC never; n (%)* | 12/20 (60%) | 74/141 (52%) | 0.6345 |
| | | | |
| *ROSC at hospital admission; n (%)* | *7/20 (35%)* | *58/141 (41%)* | |
| *Hospital admission with ongoing CPR; n (%)* | *0/20 (0%)* | *13/141 (9%)* | |
| *Death on the scene; n (%)* | *13/20 (65%)* | *70/141 (50%)* | *0.2456* |
| | | | |
| RACA-Score; Median [25th–75th] | *35 [22.75–51.25]* | *42 [30.00–61.25]* | 0.2650 |
| | | | |
| *Initial rhythm; n (%)* | | | |
| *VT/VF* | 5/20 (25%) | 43/141 (30%) | |
| PEA or asystole | *15/20 (65%)* | *98/141 (70%)* | 0.7953 |
| | | | |
| *Witness status; n (%)* | | | |
| *Witnessed arrest* | *10/20 (50%)* | *80/141 (57%)* | |
| Unwitnessed arrest | *10/20 (50%)* | *61/141 (43%)* | *0.6342* |
| CPR = Cardiopulmonary resuscitation; EMS = Emergency medical service; OHCA = Out of Hospital cardiac Arrest; PEA = Pulseless electrical activity; RACA = ROSC-after-cardiac-arrest; ROSC = Return of spontaneous circulation; VF = Ventricular fibrillation; VT = Ventricular tachycardia | | | |

**Table A.5.b**

Baseline characteristics and outcome parameters of OHCA scenarios in which CPR was initiated by a mobile responder or by EMS in patients between 70 and 79 years.

| | *Mobile responder-initiated CPR* | *EMS-initiated CPR* | *p-value* |
|---|---|---|---|
| ***Patients 70-79 years, n*** | *25* | *183* | |
| ROSC ever; n (%) | *12/25 (48%)* | *80/183 (43%)* | |
| | *13/25 (52%)* | *103/183 (57%)* | *0.8305* |
| | | | |
| *ROSC at hospital admission; n (%)* | *9/25 (36%)* | *62/183 (34%)* | |
| *Hospital admission with ongoing CPR; n (%)* | 1/25 (4%) | 16/183 (9%) | |
| *Death on the scene; n (%)* | *15/25 (60%)* | *105/183 (57%)* | 0.7187 |
| | | | |
| *RACA-Score; Median [25th–75th]* | 38 [29.50–55.50] | 36 [24.75–49.25] | 0.2906 |
| | | | |
| *Initial rhythm; n (%)* | | | |
| *VT/VF* | *6/25 (24%)* | *36/183 (20%)* | |
| *PEA or asystole* | 19/25 (76%) | 147/183 (80%) | 0.6003 |

| | | | |
|---|---|---|---|
| *Witness status; n (%)* | | | |
| *Witnessed arrest* | *14/25 (56%)* | *107/183 (58%)* | |
| *Unwitnessed arrest* | 11/25 (44%) | 76/183 (42%) | 0.8317 |

CPR = Cardiopulmonary resuscitation; EMS = Emergency medical service; OHCA = Out of Hospital cardiac Arrest; PEA = Pulseless electrical activity; RACA = ROSC-after-cardiac-arrest; ROSC = Return of spontaneous circulation; VF = Ventricular fibrillation; VT = Ventricular tachycardia

**Table A.5.c**

Baseline characteristics and outcome parameters of OHCA scenarios in which CPR was initiated by a mobile responder or by EMS in patients over 80 years.

| | *Mobile responder-initiated CPR* | *EMS-initiated CPR* | *p-value* |
|---|---|---|---|
| ***Patients over 80 years*** | *33* | *236* | |
| *ROSC ever; n (%)* | *6/33 (18%)* | *70/236 (30%)* | |
| *ROSC never; n (%)* | 27/33 (82%) | 166/236 (70%) | 0.2168 |
| | | | |
| *ROSC at hospital admission; n (%)* | 4/33 (12%) | 53/236 (22%) | |
| *Hospital admission with ongoing CPR; n (%)* | 0/33 (0%) | 11/236 (5%) | |
| *Death on the scene; n (%)* | *29/33 (88%)* | *172/236 (73%)* | *0.1445* |
| | | | |
| *RACA-Score; Median [25th–75th]* | *27 [19.00–41.50]* | *28 [19.00–40.50]* | *0.8615* |
| | | | |
| Initial rhythm; n (%) | | | |
| *VT/VF* | *5/33 (15%)* | *33/236 (14%)* | |
| *PEA or asystole* | *28/33 (85%)* | *203/236 (86%)* | *0.7933* |
| | | | |
| Witness status; n (%) | | | |
| *Witnessed arrest* | *15/33 (45%)* | *118/236 (50%)* | |
| *Unwitnessed arrest* | *18/33 (55%)* | *118/236 (50%)* | *0.7113* |

CPR = Cardiopulmonary resuscitation; EMS = Emergency medical service; OHCA = Out of Hospital cardiac Arrest; PEA = Pulseless electrical activity; RACA = ROSC-after-cardiac-arrest; ROSC = Return of spontaneous circulation; VF = Ventricular fibrillation; VT = Ventricular tachycardia

**Table A.6**

Outcome parameters of OHCA events in which CPR was initiated by a mobile responder or by EMS in index patients.

| | *Mobile responder-initiated CPR* | *EMS-initiated CPR* | *p-value* |
|---|---|---|---|
| Proportion of index patients in all patients | *23/101 (23%)* | *131/751 (17%)* | *0.2144* |
| *ROSC ever; n (%)* | *17/23 (74%)* | *99/131 (76%)* | |
| *ROSC never; n (%)* | *6/23 (26%)* | *32/131 (24%)* | *>0.9999* |
| | | | |
| *ROSC at hospital admission; n (%)* | *16/23 (70%)* | *86/131 (66%)* | |
| *Hospital admission with ongoing CPR; n (%)* | *3/23 (13%)* | *21/131 (16%)* | |
| *Death on the scene; n (%)* | 4/23 (17%) | 24/131 (18%) | 0.9201 |
| | | | |
| *RACA-Score; Median [25th–75th]* | *61.5 [58.75 - 66.25]* | *65 [62.00 - 70.00]* | 0.0406 |

*CPR = Cardiopulmonary resuscitation; EMS = Emergency medical service; OHCA = Out of hospital cardiac arrest; PEA = Pulseless electrical activity; RACA = ROSC-after-cardiac-arrest; ROSC = Return of spontaneous circulation*
